# Conserved Structural Domains in FoxD4L1, a Neural Forkhead Box Transcription Factor, Are Required to Repress or Activate Target Genes

**DOI:** 10.1371/journal.pone.0061845

**Published:** 2013-04-16

**Authors:** Steven L. Klein, Karen M. Neilson, John Orban, Sergey Yaklichkin, Jennifer Hoffbauer, Kathy Mood, Ira O. Daar, Sally A. Moody

**Affiliations:** 1 Department of Anatomy and Regenerative Biology, George Washington University School of Medicine and Health Sciences, Washington, DC, United States of America; 2 Institute for Bioscience and Biotechnology Research, Department of Chemistry and Biochemistry, University of Maryland, Rockville, Maryland, United States of America; 3 Penn Center for Bioinformatics, University of Pennsylvania, Philadelphia, Pennsylvania, United States of America; 4 Laboratory of Cell and Developmental Signaling, NIH, NCI-Frederick, Frederick, Maryland, United States of America; University of Colorado, Boulder, United States of America

## Abstract

FoxD4L1 is a forkhead transcription factor that expands the neural ectoderm by down-regulating genes that promote the onset of neural differentiation and up-regulating genes that maintain proliferative neural precursors in an immature state. We previously demonstrated that binding of Grg4 to an Eh-1 motif enhances the ability of FoxD4L1 to down-regulate target neural genes but does not account for all of its repressive activity. Herein we analyzed the protein sequence for additional interaction motifs and secondary structure. Eight conserved motifs were identified in the C-terminal region of fish and frog proteins. Extending the analysis to mammals identified a high scoring motif downstream of the Eh-1 domain that contains a tryptophan residue implicated in protein-protein interactions. In addition, secondary structure prediction programs predicted an α-helical structure overlapping with amphibian-specific Motif 6 in *Xenopus*, and similarly located α-helical structures in other vertebrate FoxD proteins. We tested functionality of this site by inducing a glutamine-to-proline substitution expected to break the predicted α-helical structure; this significantly reduced FoxD4L1’s ability to repress *zic3* and *irx1*. Because this mutation does not interfere with Grg4 binding, these results demonstrate that at least two regions, the Eh-1 motif and a more C-terminal predicted α-helical/Motif 6 site, additively contribute to repression. In the N-terminal region we previously identified a 14 amino acid motif that is required for the up-regulation of target genes. Secondary structure prediction programs predicted a short β-strand separating two acidic domains. Mutant constructs show that the β-strand itself is not required for transcriptional activation. Instead, activation depends upon a glycine residue that is predicted to provide sufficient flexibility to bring the two acidic domains into close proximity. These results identify conserved predicted motifs with secondary structures that enable FoxD4L1 to carry out its essential functions as both a transcriptional repressor and activator of neural genes.

## Introduction

Fox transcription factors contain a highly conserved Forkhead DNA binding domain (forkhead box) consisting of three α-helices, three β-strands and two wings on either side of the third β-strand. The large Fox family is subdivided into 19 sub-families, “A” – “S”, based on sequence variation within the forkhead box [1], [2], [3], [4], [5]. These transcription factors play key roles in numerous developmental and differentiation processes in nearly every tissue, and their diverse functions are likely regulated by their tissue-specific expression and associations with co-factors and/or DNA modifying enzymes. Fox proteins can regulate transcription by activation or repression; as “pioneers” they also can open the chromatin structure to other proteins [6], [7], [8], [9]. It has been suggested that these different functions are due to the divergent protein sequences in the N- and C-terminal trans-regulatory domains that flank the forkhead box. Understanding the role of these flanking regions is critical for elucidating how this family of important transcription factors can perform different transcriptional activities during numerous processes.

The FoxD sub-family is present in all chordates, and is involved in the formation of mesodermal and neural tissues. For example, Ciona has a single FoxD gene that is involved in notochord induction [10]. Amphioxus has duplicated the FoxD gene, and this duplication may be related to the evolution of the head neural crest [11]. Vertebrates have four members of the FoxD sub-family, with divergent expression patterns. In mammals, chick and frog, FoxD1 is involved in the development of the dorsolateral mesoderm and kidney, and in the formation of the anterior neural plate, retina, and forebrain [12], [13], [14], [15], [16]. In frog, FoxD2 is expressed primarily in the paraxial mesoderm, migrating muscle precursors, cranial neural crest and diencephalon [17], [18]. In mouse, FoxD2 is expressed in several mesodermal derivatives including sclerotome, in the neural crest derived head mesenchyme, midbrain and forebrain [19], [20]. FoxD3 is involved in mesoderm formation at gastrula stages and later is required for neural crest development [17], [21], [22], [23], [24], [25], [26]. FoxD4 (mouse, human) and the highly related FoxD4L1 (human, fish, frog; aka FoxD5 in fish and frog) are expressed in the early neural ectoderm [27], [28], [29], [30], [31], [32], [33], [34], [35]; in zebrafish, FoxD4L1 also is expressed in the mesoderm and plays a role in somitogenesis [36]. In frog (*Xenopus laevis*), FoxD4L1 plays a key role in regulating the expression of at least 11 other neural ectodermal transcription factors (neTFs) induced by the neural inductive signaling that occurs during gastrulation [34], [37].

Knock-down of *Xenopus* FoxD4L1 reduces the expression of all 11 neTF genes, showing that it acts up-stream, consistent with potential Fox binding sites in the proximal upstream region of each gene [37]. Increasing FoxD4L1 expression within the neural plate showed that this single transcription factor both represses and activates targets. It down-regulates genes in the BMP signaling pathway, epidermal genes and neTF genes that initiate neural differentiation, and it up-regulates neTF genes that maintain an immature, proliferative neural ectoderm [34], [37], [38]. Thus, FoxD4L1 mediates the transition of neural ectoderm to neural stem cells by controlling the balance between transcription factors that promote proliferation versus differentiation.

Our recent findings show that the different functions of FoxD4L1 depend upon the N- and C-terminal trans-regulatory domains that flank the forkhead box. Its repressive ability depends upon the C-terminus, within which is an Engrailed homology region-1 [Eh-1] that can bind the co-repressor protein, Groucho [Grg in vertebrates; TLE in humans] [39]. This domain is found in several Fox proteins, including all members of the FoxD sub-family (reviewed in [4], [34], [40]). In FoxD3, FoxA1 and FoxA2, Grg binding to the Eh-1 motif plays an important role in repressing downstream targets [41], [42]. Our studies showed that Grg4 binding enhances FoxD4L1 repressive activity, particularly when FoxD4L1 is present at low concentrations, but it does not account for all of the repressive activity [39]. Herein, we identify additional sites that are predicted to contribute to FoxD4L1’s repressive activity. We experimentally demonstrate that one of these sites (Motif 6), which is predicted to form an α-helix, contributes to neural target gene repression independent of Grg4 binding.

The activating ability of FoxD4L1 depends upon a 14 amino acid “acidic blob” region (AB) in the N-terminus [39]; in *Xenopus* ABs are only found in the FoxD sub-family [4]. Within the AB are four highly conserved amino acids, predicted to form a β-strand, that separate two acidic domains. Disrupting this region indicates that the β-strand is dispensable for target gene activation, but a glycine residue, which is predicted to provide sufficient flexibility to bring the two acidic domains into close proximity, is required. These findings indicate that conserved regions flanking the forkhead box contain predicted motifs and secondary structure that enable FoxD4L1 to function as both a repressor and activator.

## Materials and Methods

This study was carried out in strict accordance with the recommendations in the Guide for the Care and Use of Laboratory Animals of the National Institutes of Health. The protocol was approved by the IACUC of the George Washington University (#A-3205) and the IACUC of the NCI (#12-433). All surgery was performed under tricaine-methane sulfonate anesthesia, and all efforts were made to minimize suffering.

### Protein structure prediction analyses

FoxD4/FoxD4L1 sequences were retrieved from Ensembl database 69 (ensembl.org) based on chromosome synteny and sequence homology. Accession numbers of FoxD4/FoxD4L1 sequences used in this analysis are provided in Table S1. Multiple sequence alignments were constructed using T-COFFEE, version 7.7.1. (tcoffee.vital-it.ch/cgi-bin/Tcoffee/tcoffee_cgi/index.cgi [43]. Aligned FoxD4/FoxD4L1 proteins were edited using BioEdit Sequence Alignment Editor version 7.0.4.1. [44]. The expectation-maximization algorithm of the MEME program (Multiple Em for Motif Elicitation, Version 4.9.0) was used to identify potential functional and regulatory motifs in the C-terminus on the server (hmeme.nbcr.net/meme/). The search parameters used were 6–8 motifs per a run and a motif size of 8–15 amino acid residues. The protein sequences of FoxD4/FoxD4L1 also were analyzed for the presence of canonical leucine zippers using server (2zip.molgen.mpg.de/) [45]. Finally, the prediction of secondary FoxD4L1A (*Xenopus laevis*) structure was conducted using Psipred [46], [47], Porter [48] and a consensus secondary structure prediction on the server: npsa-pbil.ibcp.fr/cgi-bin/npsa_automat.pl?page = /NPSA/npsa_seccons.html. The helical wheel was modulated using the sequence FoxD4L1A (313–330 aa) on the server cti.itc.virginia.edu/∼cmg/Demo/wheel/wheelApp.html. All analyses were repeated at least 3 times.

### Creation of mutant FoxD4L1 plasmids

We deleted and mutated sites in Myc-tagged-*foxD4L1* in the pCS2+ vector using the Quik-change mutagenesis kit (Stratagene). The C-terminal mutations were made using the following primers and their complements: 5′-CTGGCCCTCTGGCAGCCAATACTC-3′ for the L to A substitution; 5′-AGCCAATACTCGGGGTGCCAGGC-3′ for the Q to R substitution; 5′CAGGGTGCCAGGGGATACAACCTCATAC-3′ for GARG; and 5′-CAGGGTGCCAGGCCATACAACCTCATA-3′ for GARP. The N-terminal mutations were generated using the following primers and their complements: 5′-GATGAGGAGGATGAAGATGATCCCTGCAGC-3′ for the AB1 deletion; 5′-GATCATCTTCTCCTGCAGCGGCCGCAGCTGCTTCATCCTCCTC-3′ for AB2; and 5′-GAGGAGGATGAAGCAGCTGCGGCCGCAGCAGATGATCCCTGC-3′ for AB4. All mutagenesis reactions were performed with an annealing temperature of 55°C. Mutant FoxD4L1 inserts generated in pCS2+MT were excised with Stu1/Asp718 and subcloned into pCS2+.

### mRNA synthesis and injection

mRNAs encoding *foxD4L1* mutant proteins were synthesized *in vitro* (Ambion, mMessage mMachine kit). These mRNAs (100 pg/nl each) were mixed with nuclear localized *βgal* mRNA (100 pg/nl) as a lineage tracer. Embryos were obtained, cultured and microinjected as previously described [49], [50]. One nl of each mRNA mixture was microinjected into a defined precursor of the neural ectoderm (blastomere D1.1) [51] on one side of the 16-cell embryo. This results in FoxD4L1 protein expression in about 50% of the neural plate only on the experimental side of the embryo, ensuring that the mutant protein does not disrupt earlier morphogenesis and avoiding non-specific effects or embryonic lethal phenotypes. The uninjected side of the embryo was used as an internal control. In some experiments, mutant *foxD4L1* mRNA (plus *βgal* mRNA) was injected into a defined precursor of the non-neural epidermis (blastomere V1.1) [51] to test for its ability to ectopically induce neTF gene expression.

### Whole embryo in situ hybridization

Embryos were cultured to Nieuwkoop and Faber [52] stages 10.5–12.0 (for *gem*, *zic2*, *zic3*) and 13/14 (for *irx1*), and processed for in situ hybridization (ISH) as previously described [53]. Anti-sense Dig-labeled RNA probes were synthesized as previously described [37]. The expression patterns of *gem*, *zic2, zic3*, and *irx1* were compared on the experimental and control sides of embryos derived from at least three different clutches of eggs from different sets of adult parents to account for population variability. The frequency at which embryos showed altered expression was compared to the frequency from wt-FoxD5-injected samples using the Chi-squared statistic (p<0.001).

### Western blots and Co-IPs

To ensure that mutant proteins were translated, oocytes were surgically removed from female frogs using standard techniques [54]. Oocytes were subjected to enzymatic defolliculation in 5 mg/ml collagenase type IV (Sigma), staged according to established procedures [54] and maintained in 1X Modified Barth’s Solution (MBS: 5 mM HEPES pH 7.8, 88 mM NaCl, 1 mM KCl, 0.7 mM CaCl_2_, 1 mM MgSO_4_, 2.5 mM NaHCO_3_) at 18°C overnight. Mature oocytes were injected with 5 ng of mRNAs coding for myc-tagged wt-FoxD4L1 or myc-tagged mutants of FoxD4L1 and cultured overnight at 18°C. Oocytes were lysed in HNTG (20 mM HEPES pH 7.4, 150 mM NaCl, 1.5 mM MgCl_2_, 1 mM EGTA, 10% glycerol, 1% TritonX-100) containing a protease inhibitor cocktail (CalBiochem) and 1 mM PMSF, 3 mM β-glycerolphosphate and 4 mM Na Vanadate. 15 µl (1.5 oocyte equivalents) lysate was prepared with 2X sample buffer and run on 10% Miniprotean TGX precast gels (Biorad), transferred to nitrocellulose using standard methods, and blocked in Tris-buffered saline (25 mM Tris) +0.1% Tween-20 (TBST) +5% nonfat dry milk for 1 hour at room temperature. Western blots were incubated with anti-Myc-primary antibody (Cell Signaling) at 4°C overnight, washed with TBST and incubated with an anti-mouse IgG HRP linked secondary antibody (Cell Signaling) for 1 hour at room temperature. Following antibody incubation, blots were rinsed with TBST, blotted with a chemiluminescent HRP antibody detection reagent (Pierce ECL Substrate) and exposed to film.

For Co-IP analyses, oocytes were injected with 5 ng of either myc-tagged wt-FoxD4L1 or myc-tagged C-terminal mutants of FoxD4L1 and/or HA-tagged Grg4 and incubated as above. For each immunoprecipitation reaction, 150 µl of lysate (15 oocyte equivalents) was mixed with 650 µl ice-cold TNSG lysis buffer and 1 µg of antibody (raised against HA or Flag; Applied Biological Materials) and incubated at 4°C for 1–2 hours, after which 25 µl protein A/G agarose beads (Santa Cruz Biotechnology) were added to the reaction and rotated in an orbital mixer overnight at 4°C. Beads were briefly pelleted at 4°C and rinsed 3 times with ice-cold TNSG lysis buffer. All residual buffer was removed with a flat pipette tip and beads were resuspended in 45 µl 1X RIPA sample buffer (RIPA Buffer: 150 mM NaCl, 1% NP40, 0.5% Na Deoxycholate, 0.1% SDS, 50 mM Tris (8.0); 4X sample buffer: 4 mL 10% SDS, 2 mL glycerol, 0.3086 g DTT, 0.00001 g Bromphenol Blue; 4X sample buffer was diluted to 1X in RIPA buffer). Samples were boiled at 100°C for 10 minutes prior to loading on Tris-glycine SDS-Polyacrylamide 10% gels. Proteins were resolved by SDS/PAGE, as described above.

### Immunostaining

To ascertain whether the two mutant FoxDL1 proteins that did not display normal function had access to the nucleus, dorsal blastomeres were injected with *myc*-tagged *AB4* or myc-tagged *GARP* mRNAs and embryos fixed at stages 12–13 in 4% paraformaldehyde in PBS. Frozen sections were cut with a cryostat and subjected to standard immunofluorescence staining protocols using an anti-Myc-tag primary antibody (#9B11, Cell Signaling Tech., 1∶2000), a goat anti-mouse IgG Alexa Fluor 488 conjugated secondary antibody (#4408, Cell Signaling Tech., 1∶1000) followed by counterstaining of the nuclei with DAPI. Images were collected using a Zeiss LSM 710 confocal system equipped with 32-channel spectral photomultiplier. Thirty-two channel spectral stacks were collected at spectral resolution of 9.6 nm within the range of 418 – 726 nm. To obtain the signature spectral curves of autofluorescence, DAPI and Alexa Fluor 488 emissions, spectral confocal images were taken with excitation of either the 405 nm diode laser (DAPI and autofluorescence) or the argon 488 laser line (Alexa Fluor 488); these spectral curves were then used to unmix the DAPI, autofluorescence and Alexa Fluor 488 emissions registered upon simultaneous excitation of the samples with 405 and 488 laser lines.

## Results

### Identification of potential repressive motifs in the C-terminal regions of FoxD4/FoxD4L1 proteins

**Figure 1:**
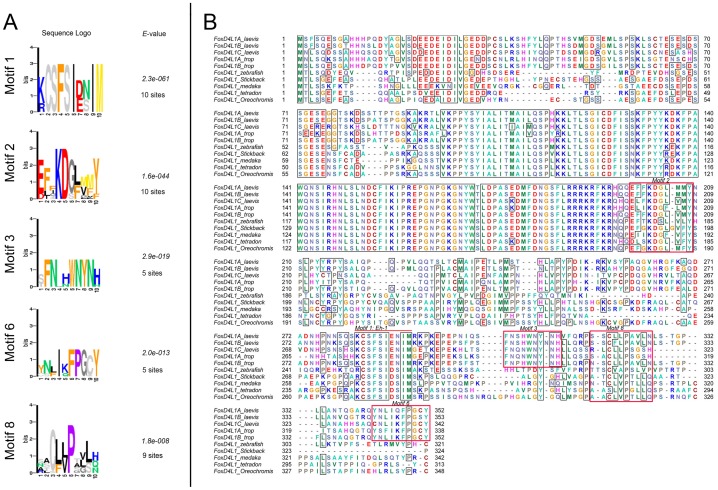
Five statistically significant C-terminal motifs identified with the expectation-maximization algorithm implemented in the MEME program [**55**]. (A) Five identified statistically significant motifs in amphibian and fish FoxD4L1 sequences. “Sites” indicates how many sequences contain the indicated sequence logo. (B) Selected identified motifs from (A) are outlined in red on the FoxD4L1 sequence alignment. Motif 1, the Eh-1 motif is indicated by a red bar.

We previously reported that although the ability of *Xenopus* FoxD4L1 to down-regulate *zic* and *irx* genes involves the binding of the Grg4 co-repressor to the Eh-1 motif in the carboxyl (C-) region of the protein, there also is an unidentified site(s) towards the C-terminus that contributes to repression [39]. To identify potential functional peptide motifs in the C-terminus of *Xenopus* FoxD4L1A-related sequences, a multiple sequence alignment of FoxD4L1 of the closely related fish and amphibians was constructed (Figure S1). This sequence set was further analyzed for the presence of statistically significant motifs using the expectation-maximization algorithm implemented in the MEME program [55]. The N-terminal domain, the forkhead DNA-binding domain, and a putative nuclear localization signal (NLS) were excluded from the sequences analyzed. Based on the search parameters, the analysis identified 8 motifs: 5 motifs were common for both fish and amphibian FoxD4L1 and 3 were amphibian specific. The motifs are enumerated from 1 to 8 based on the score of the *E*-value (Figure S2). The sequence logos of the motifs with a strict (non-divergent) sequence pattern are shown on Figure 1A and outlined in red on the sequence alignment in Figure 1B. As expected, the highest scoring motif (*E* = 2.3e-061) is an Eh-1 motif (*Xenopus* FoxD4L1A aa282–291), which is known to be a Grg4-interacting sequence [39]. Motif 2 (*Xenopus* FoxD4L1A aa199–205; *E* = 1.7e-044) is located upstream of the Eh-1 motif near the putative NLS sequence and is conserved between fish and amphibian FoxD4L1 sequences. Motif 3 (FoxD4L1A aa303–311; *E* =  2.9e-019) is located C-terminal to the Eh-1 motif, and is present only in the amphibian FoxD4L1 sequences. Motif 6 (FoxD4L1A aa342-352; *E* =  2.0e-013) is found at the extreme C-terminus, and Motif 8 (FoxD4L1A aa318-327; *E* =  1.8e-008) is found between Motif 3 and Motif 6. This analysis thus identified novel specific motifs (Figure S2) with high *E*-values, some of which are conserved between fish and amphibian FoxD4L1, in the C-terminal region that our previous deletion study indicated is involved in repressive activity [39].

**Figure 2:**
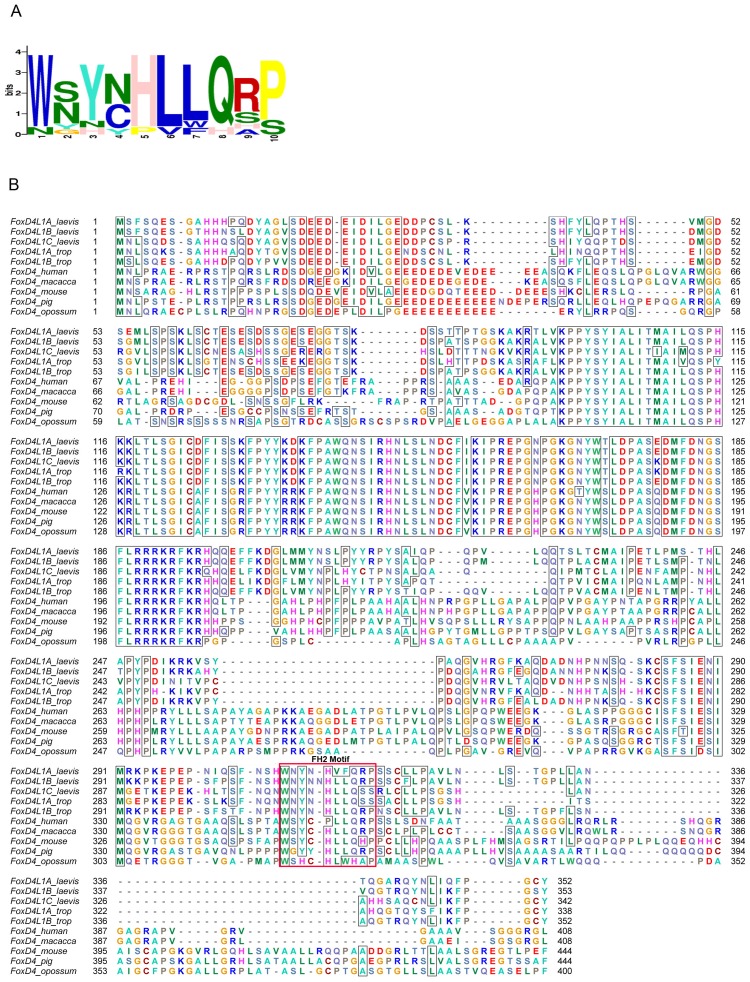
The C-terminus of FoxD4/FoxD4L1 from frog and mammals contains a novel specific conserved motif, which we term the Fox homology motif 2 (FH2). (A) The sequence logo of the 10 amino acid FH2 motif. (B) The FH2 motif is outlined in red on the FoxD4/FoxD4L1 sequence alignment.

Next, multiple sequence alignments of FoxD4L1 of amphibians and mammals were constructed to reveal conserved C-terminal regions that might have formed as novel motifs in tetrapods. A similar analysis was conducted as described above using the MEME search. In addition to identifying the Eh-1 motif, the MEME search identified a second scoring motif (Figure 2A; aa 308–318; *E* = 1.3e-034) located downstream of the Eh-1 motif. This motif overlaps with the previously identified Motif 3 (Figure 1B). High scoring of this motif, which we term the Fox homology motif 2 (FH2), is consistent with its evolutionary conservation in the FoxD4/FoxD4L1 proteins of mammals and amphibians, which generally share low homology within the C-terminus (Figure S3), and suggests functional relevance. It is notable that the FH2 motif contains several aromatic residues, including a highly conserved tryptophan residue, (*Xenopus* FoxD4L1A, 308 aa), shared between amphibian and mammalian FoxD4/FoxD4L1 proteins. In some functional motifs of transcriptional regulators, a tryptophan residue is known to be implicated in protein-protein interactions. For example, the tryptophan residue in the motif (WACKAKRK) mediates physical interaction of MyoD with Pbx-Mes1/Prep1 [56]. In the transcription factor, Hairy, the tryptophan residue in the motif WRKY is crucial for mediating binding to the Groucho co-repressor [57]. By adapting a BLAST search for short sequences, we searched for the presence of similar sequences in other proteins; however, the search did not result in the identification of specific similar sequences in other transcription factors. This may indicate that the FH2 motif could be FoxD4/FoxD4L1 sub-family specific. A number of other motifs were identified in this analysis (Figure S4), the majority of which were *Xenopus* specific and similar to those previously identified (Figure S1).

We also noted that the C-terminus of the FoxD4/FoxD4L1 proteins analyzed contains repetitive leucine residues, overlapping with the FH2 motif, that have the following pattern: ([L/V][L/F/W]XXXXXX[L/F]LXX[L/V]LX[L/M]), (*Xenopus* FoxD4L1A, 313–334 aa). This repeat resembles a relaxed leucine zipper pattern found in other transcription factors [58]. We subjected this sequence to an algorithm implemented in the program 2ZIP [45], but this analysis did not identify a canonical leucine zipper. Therefore, we conducted the helical wheel modeling to reveal amphipathicity of this region using Val 313 as a stem residue. The wheel model revealed that the hydrophobic surface of the predicted helix and is a surface consisting predominantly of hydrophilic residues (Figure S5), which may indicate that the region can form amphipathic helical regions.

Finally, we ran predictions of secondary structure of *Xenopus* FoxD4L1A. Using consensus secondary prediction, which includes a majority of algorithms for the prediction of secondary structure via the Network Protein Sequence server, we confirmed the secondary structure of the forkhead box (FRK); combined algorithms predict the majority of the helical structure (Figure S6) compared to the crystal structural data on the related FoxD3 FRK [59]. Some algorithms predict secondary structure in the leucine repetitive region, which is consistent with amphipathicity of this region, and a sheet region for the FH2 motif. Additionally, helical structure is predicted for the near C-terminus sequence GARQYNLIQFPG (aa339–350), which overlaps with Motif 6 (Figure 1). Porter also predicted a short α-helical segment in this sequence (aa 339–345, GARQYNLI), although Psipred predicted this region to be random coil (Table 1). Mouse and human FoxD4/FoxD4L1 proteins also are predicted by Psipred and Porter to have α-helices in this region (Table 1), suggesting it has functional significance.

**Table 1:** Predicted structures in FoxD proteins.

	N-terminal β-strand			C-terminal α-helix		
		Psipred	Porter	Eh1	Psipred	Porter
Xenopus FoxD1	IDVV, aa 17–20; G = aa21	random coil	random coil	aa 294–300	aa 327–331	aa 325–332
Xenopus FoxD2	IDVV, aa19–22; G = aa24	random coil	aa 21–22	aa 276–282	random coil	random coil
Xenopus FoxD3	IDVV, aa25–28; G = aa29	random coil	aa 27–28	aa 297–303	aa 361–366	aa 361–366
Mouse FoxD3	IDVV, aa25–28; G = aa29	random coil	aa 25–28	aa 362–368	aa 447–460	aa 448–459
Xenopus FoxD4L1	IDIL, aa 26–29; G = aa30	random coil	aa 26–29	aa 285–291	random coil	aa 339–345
Human FoxD4L1	IDVL, aa27–30; G = aa31	random coil	aa 27–30	aa 324–330	aa 367–370; aa 398–401	aa 380–383; coil
Human FoxD4	IDVL, aa27–30; G = aa31	random coil	aa 27–30	aa 327–334	aa 356–365; aa 389–401; aa 430–435	aa 364–367; aa 388–400; aa 431–434
Mouse FoxD4	IDVL, aa27–30; G = aa36	random coil	aa 27–30	aa 320–326	aa 411–418; aa 428–434	aa 411–419; coil

Legend: The N-terminus of each FoxD protein contains the conserved IDVV/IDIL/IDVL sequence at the amino acid (aa) location indicated, closely followed by a glycine (G) residue. Psipred predicts these regions to be random coil, whereas Porter predicts most of them to form a β-strand at the amino acids indicated. The C-terminus of each FoxD protein contains a conserved Eh-1 motif at the amino acid (aa) location indicated. At locations downstream of this motif, the proteins are predicted to either be random coil or to form an α-helical structure at the indicated locations.

### The C-terminal α-helix in Xenopus FoxD4L1A protein contributes to target neural gene repression

**Figure 3:**
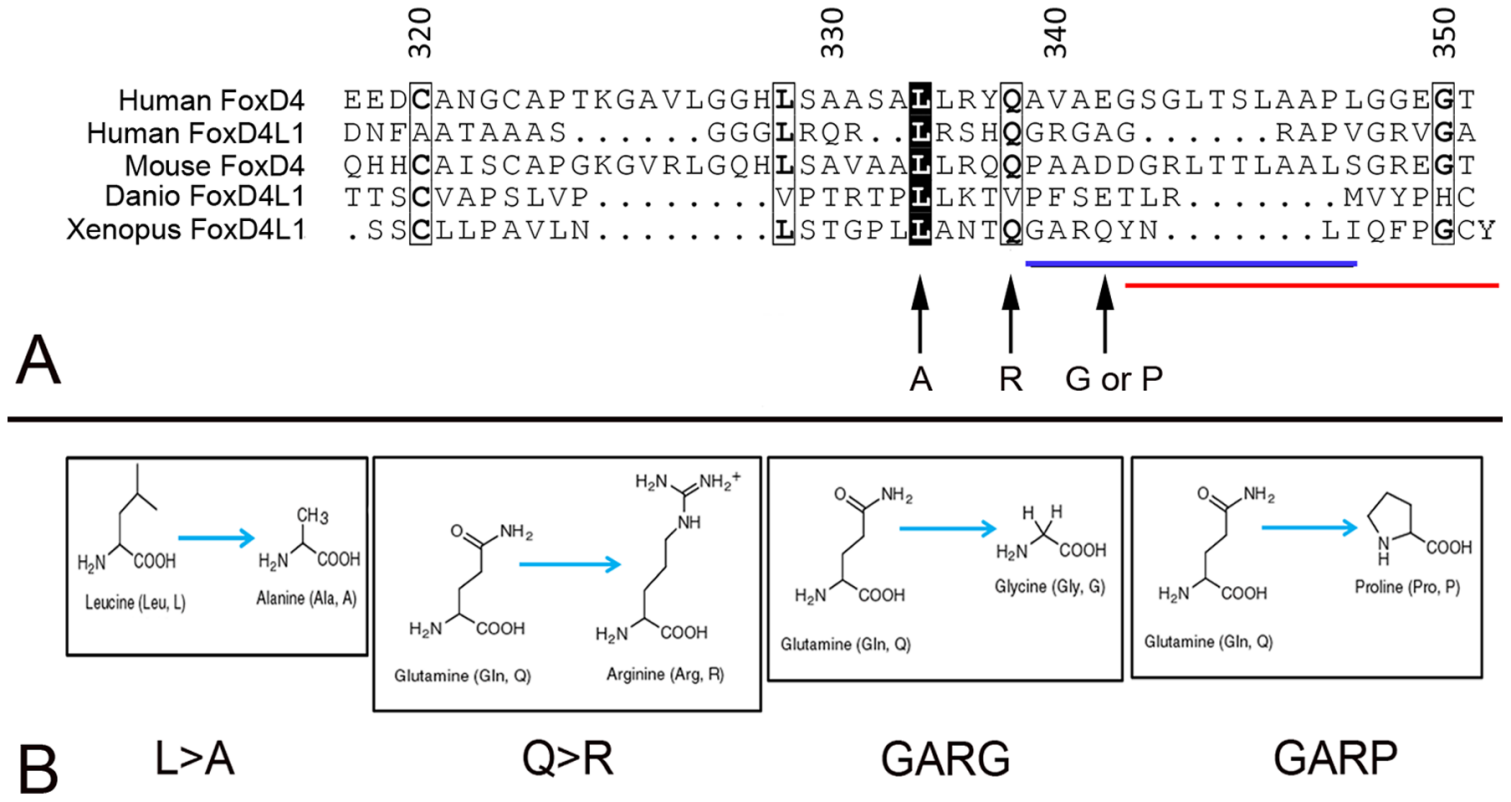
Conserved amino acids in the extreme C-terminus of FoxD4/FoxD4L1 proteins. (A) CLUSTALW alignment [64], viewed in ESPript [65], of the extreme C-terminal region of human FoxD4 (UniProtKB/Swiss Prot accession number Q12950), human FoxD4L1 (Q9NU39), mouse FoxD4 (Q60688), *Danio* FoxD4L1 (O73784) and *Xenopus laevis* FoxD4L1 (Q9PRJ8). The black boxes highlight identical amino acids, the light boxes highlight conserved amino acids and the bold letters indicate identical amino acids within a conserved region. The blue line denotes the amino acids in the *Xenopus* sequence predicted to form an α-helix, and the red line denotes Motif 6 (Fig. 1A). Arrows denote amino acid substitutions in the C-terminal mutants used in this study (L>A; Q>R; GARQ>GARG; GARQ>GARP). (B) Amino acid changes made in the C-terminal mutants used in this study.

**Figure 4:**
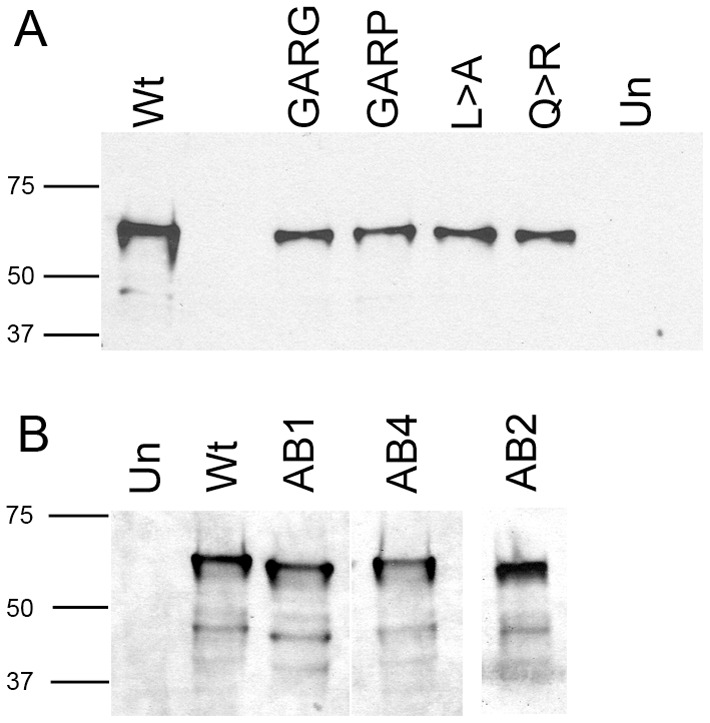
Mutant FoxD4L1 proteins are adequately expressed. Western blots of lysates from oocytes injected with mRNAs encoding C-terminus mutants (A) or Acidic Blob mutants (B) show expression of each mutant protein. Un, lysates from uninjected oocytes; Wt, lysates from wild-type FoxD4L1 injected oocytes.

Based on this information, we tested in *Xenopus* embryos the functionality of one of the predicted repressive sites: the predicted α-helix/Motif 6 at the extreme C-terminus. We replaced the Q (aa341) with either G (GARG, predicted to destabilize an α-helix) or P (GARP, predicted to disrupt an α-helix) (Figure 3A). CLUSTALW alignment of five vertebrate FoxD4/FoxD4L1 proteins identified two highly conserved amino acids just upstream of the predicted α-helix (L, aa334; Q, aa338; Figure 3A). Therefore, we designed mutant FoxD4L1 constructs that altered the length of the side chains of these amino acids (L>A; Q>R; Figure 3A) to potentially destabilize the adjacent predicted α-helix. Western blots of myc-tagged versions of these mutants demonstrate that the mRNAs each produce abundant protein (Figure 4A).

**Figure 5:**
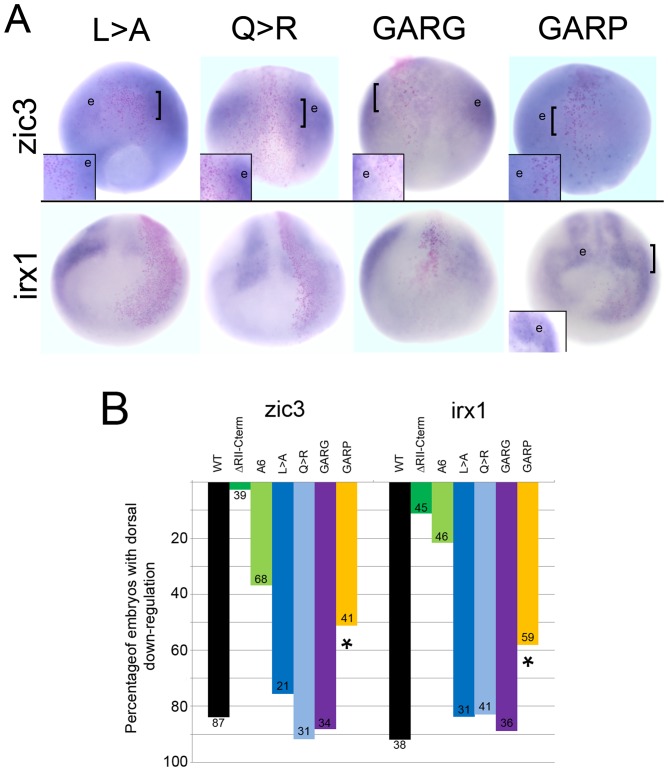
The ability to down-regulate *zic3* and *irx1* is lost in the GARP mutant. (A) The FoxD4L1 C-terminal mutant expressing clones, marked by nuclear β-Gal (pink dots), are located in the neural ectoderm. For L>A, Q>R and GARG mutants, the βGal labeled cells are less intensely stained (blue) than their neighboring cells (e) expressing endogenous levels of *zic3* or *irx1*. For GARP, the βGal labeled cells are stained at the same intensity as the neighboring cells (e). Insets are higher magnifications of the clone, the position of which is indicated on the whole embryo by a bracket. For *zic3*, images are dorsal views with vegetal pole towards the bottom; for *irx1*, images are frontal views with dorsal towards the top. (B) The percentage of embryos in which the FoxD4L1 C-terminal mutants caused down-regulation of *zic3* or *irx1* in the dorsal neural ectoderm. Numbers on each bar indicates sample size; * indicates significant difference from wild type (WT) at the p<0.001 level. Data for WT, ΔRII-Cterm and A6 are from [39].

**Figure 6:**
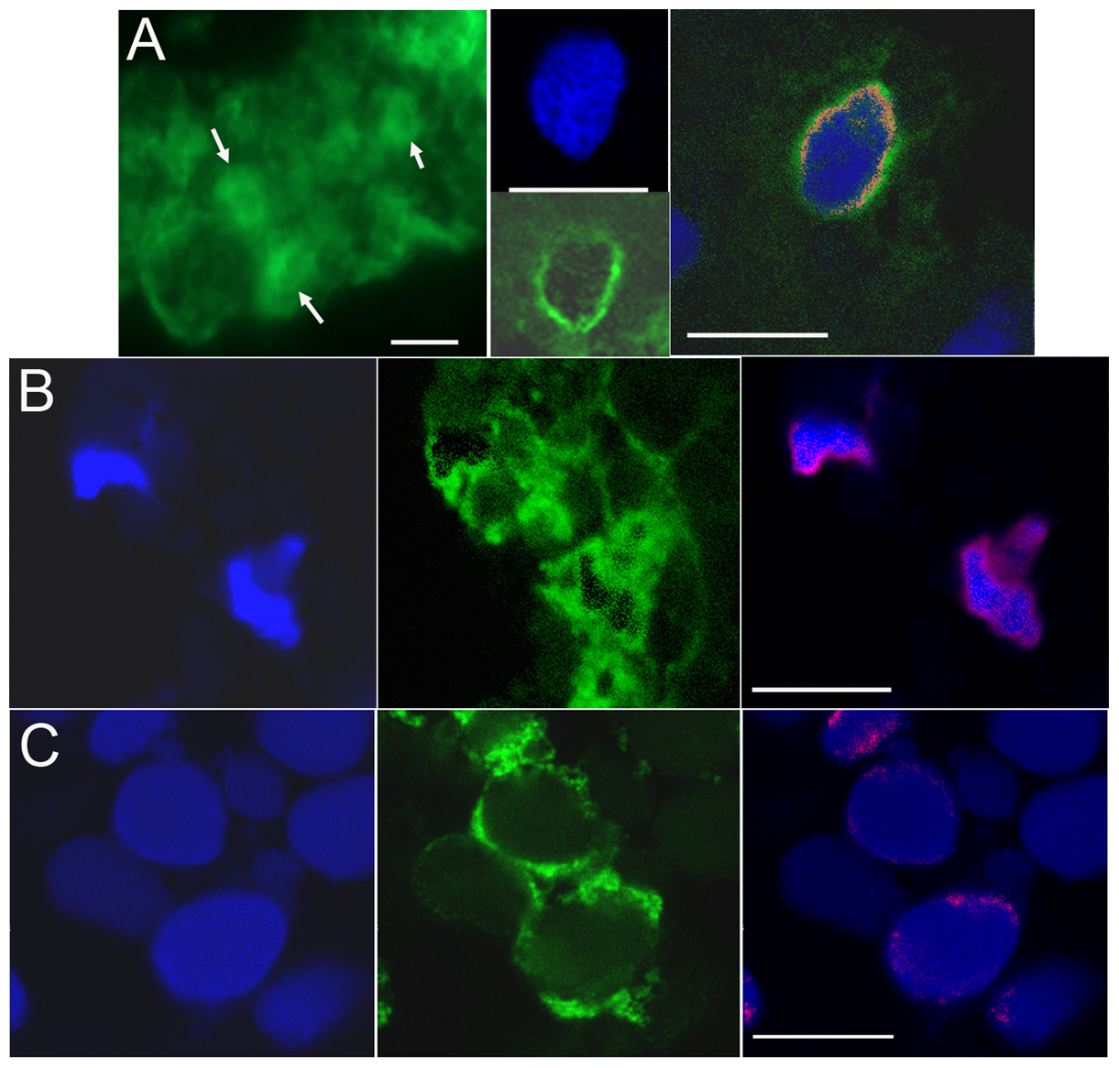
FoxD4L1 mutant proteins have access to the nucleus in a pattern similar to wild-type FoxD4L1. (A) Left panel: epifluorescence image of wild-type, myc-tagged FoxD4L1 protein in neural ectoderm of stage 13 embryo. Tagged protein is in the cytoplasm and in the nucleus (arrows). Middle panel: confocal image of a similar sample shows that the protein (green) is localized in the periphery of the nucleus (blue) where chromatin is concentrated in non-mitotic cells. Right panel: example from a similar sample in which a 32-channel signature spectral curve analysis was performed. Red pixels around the periphery of the nucleus represent sites of DNA (blue) and protein (green) colocalization. (B) Left panel: DAPI nuclear staining of cells in the superficial neural ectoderm of stage 12 embryo. Middle panel: Myc-tagged GARP protein (green), like wild-type protein, is found in the cytoplasm and in the periphery of the nucleus. Right panel: a 32-channel signature spectral curve analysis was performed to demonstrate with confidence nuclear localization of the tagged protein. Magenta pixels represent sites of DNA (blue) and protein colocalization. (C) Left panel: DAPI nuclear staining of cells in the deep layer of the stage 14 neural plate. Middle panel: Myc-tagged AB4 protein (green) also is found in the cytoplasm and in the periphery of the nucleus. Right panel: a signature spectral curve analysis was performed: magenta pixels represent sites of DNA (blue) and protein colocalization. White bars indicate 7 µm.

**Figure 7:**
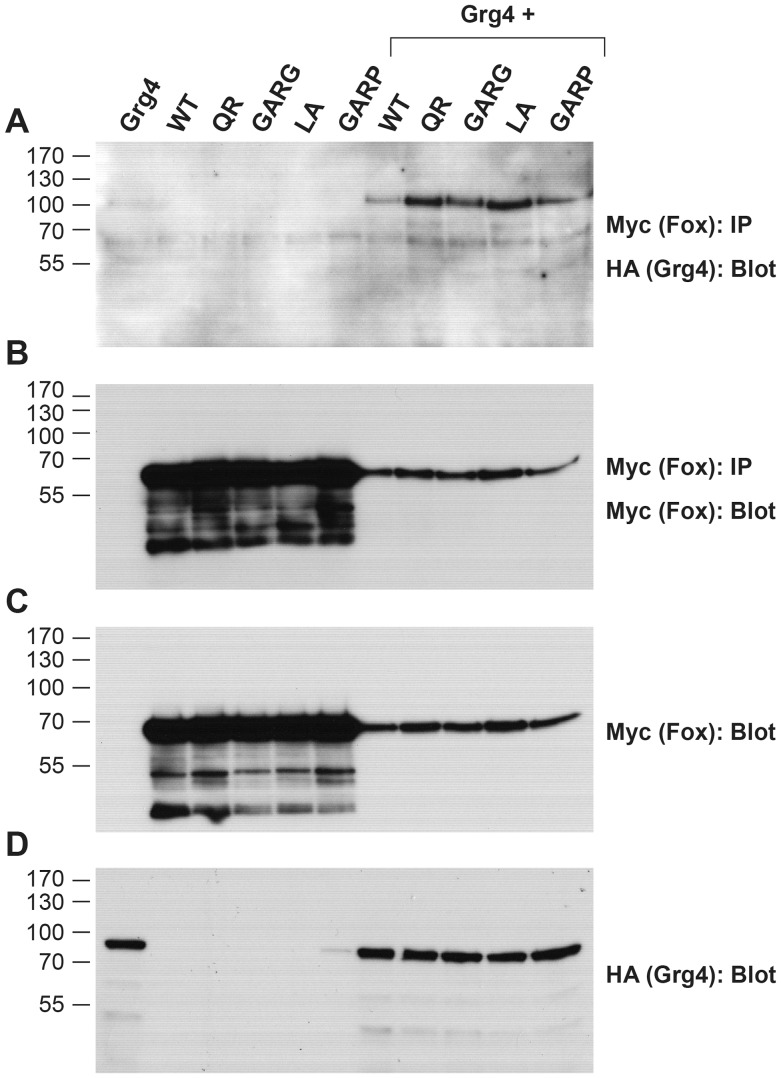
Grg4 binds to the FoxD4L1 C-term mutants. (A–D) Myc-tagged versions of wild-type (WT), as well as mutants harboring amino acid substitutions downstream of the Eh-1 domain (QR, GARG, LA, GARP) in FoxD4L1 were expressed in *Xenopus* oocytes along with HA-tagged wild-type *Xenopus* Grg4. Co-immunoprecipitation (IP) and Western blot (WB) analyses of oocyte lysates expressing HA- and Myc-tagged constructs are indicated. (A) All four constructs bind with Grg4. The control panels (B–D) show that the IPs contain similar levels of FoxD4L1 wild-type and mutant proteins (B), as do the direct lysates (C). Grg4 expressing lysates also show similar levels of this protein (D). Note: Although the co-expression of Grg4 along with the wild-type and mutant Fox constructs shows similar protein levels and binding in the IPs, it is worth noting that there is a marked reduction in expression of all Fox proteins in the presence of Grg4. This may be due to degradation, rather than competition for ribosomes that affects translation, since Grg4 levels are not affected.

These mRNAs were then expressed in a neural progenitor blastomere, and embryos analyzed for down-regulation of either *zic3* or *irx1* by in situ hybridization. The mutants in which an α-helical structure is predicted to be destabilized (L>A; Q>R; GARG) did not lose the ability to down-regulate either *zic3* or *irx1*; their repressive activity was equivalent to that described for the wild type protein [37], [39]. As shown in Figure 5A, those cells expressing L>A, Q>R, or GARG mutant FoxD4L1, which were marked by a nuclear red βgal lineage tag, expressed lower levels of *zic3* and *irx1* compared to neighboring cells; the percentage of embryos showing this repressive phenotype did not differ from those expressing the wild type protein (Figure 5B). In contrast, the construct designed to disrupt the predicted α-helical structure by replacing glutamine with proline (GARP) was significantly impaired in its ability to down-regulate *zic3* and *irx1* (Figure 5). We performed a confocal microscopic analysis of the cellular localization of a myc-tagged version of GARP protein to make sure the mutant protein could access the nucleus, and thus eliminate this as the cause for its impaired function. Wild-type, myc-tagged FoxD4L1 protein is abundant in the cytoplasm (Figure 6A), as is common for Fox proteins (e.g., see http://www.abcam.com/FOXD3-antibody-ab64807.html#description_images_2), and accumulates at the periphery of the nucleus (Figure 6A), as do the previously reported mutant FoxD4L1 proteins [39]. The same cytoplasmic and peripheral nuclear localization of the GARP protein was observed (Figure 6B). To ascertain with confidence that the GARP immunofluorescence was intra-nuclear, a 32-channel spectral analysis with resolution at 9.6 nm was performed for each excitation wavelength to eliminate autofluorescence or signal bleed-through. We then collected signals only within those signature spectral curves during simultaneous excitation with both laser lines. This analysis identified single pixels containing both signatures, which are indicated by magenta colored pixels (Figure 6B). This high resolution analysis confirms that the GARP protein has access to the nucleus. For both *zic3* and *irx1*, deleting all the amino acids from the Eh-1 motif to the end of the protein (ΔRII-C-term; Figure 5B) nearly eliminated repression [39]. In contrast, either mutating the Eh-1 motif so it can not bind Grg4 (A6) [39] or disrupting the C-terminal α-helical structure (GARP) only partially reduced repression (Figure 5B), suggesting that the repressive activities of the two regions are independent and additive. This is confirmed by the finding that the GARP mutant is able to interact with Grg4 in a co-immunoprecipitation assay (Figure 7), indicating that its repressive activity is not due to loss of Grg4 binding at the Eh-1 motif. These results indicate that both the Eh-1 domain and the α-helix/Motif 6 region participate in target neTF gene repression.

### The N-terminal Acidic Blob of FoxD4/FoxD4L1 proteins contains two acidic regions separated by four conserved amino acids that activates target neural genes

**Figure 8:**
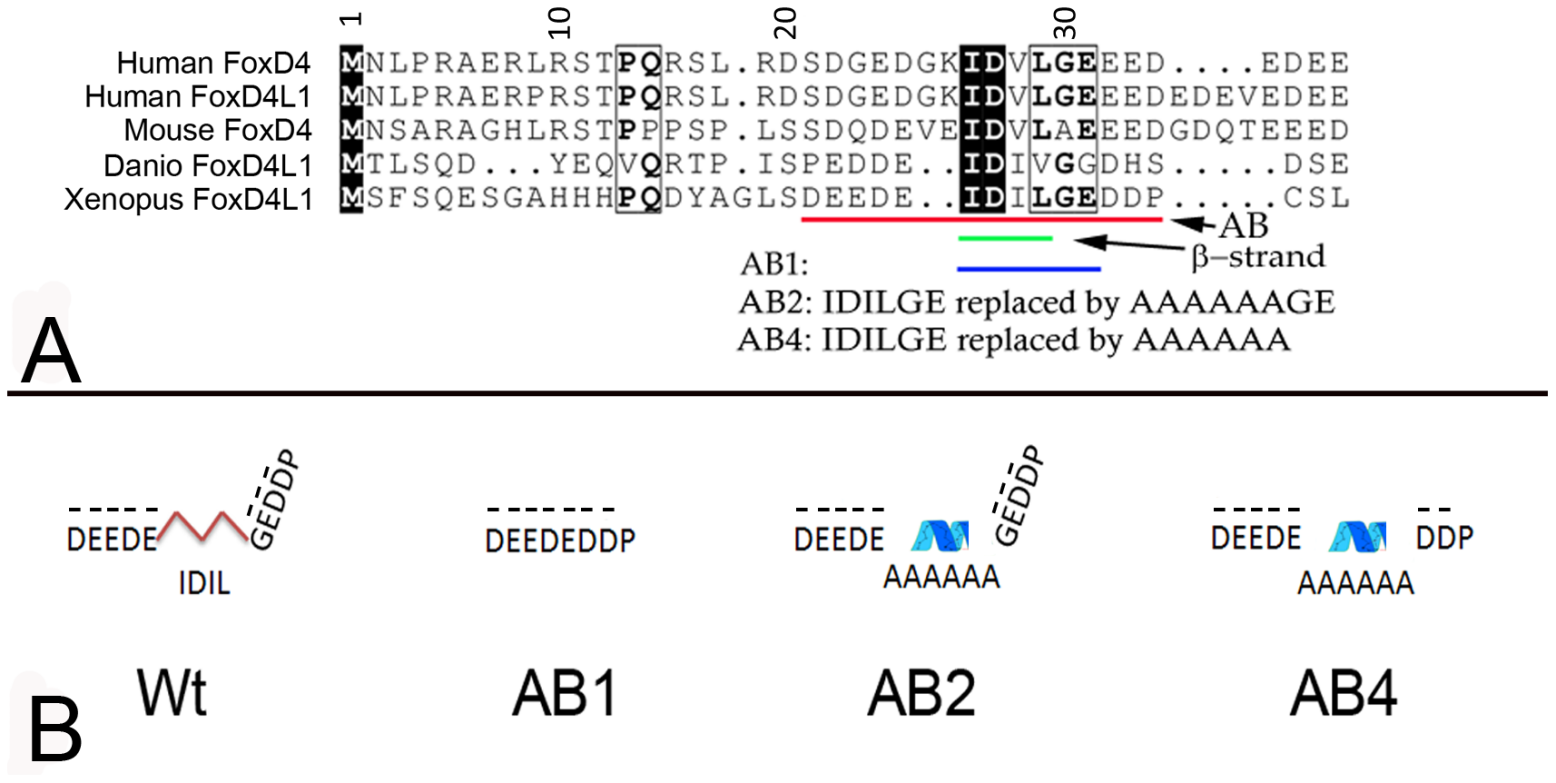
Conserved amino acids in the Acidic Blob region of FoxD4/FoxD4L1 proteins that were mutated for this study. (A) CLUSTALW alignment of the N-terminal region including the Acid Blob (AB, denoted by red line), as in Figure 3. The highly conserved IDIL sequence is predicted to form a short β-strand (green line). Six amino acids, denoted by the blue line, were deleted in the AB1 construct. The amino acid substitutions made in the AB2 and AB4 constructs are noted. (B) Predicted protein folding within the Acidic Blob of the wild-type (Wt) and AB mutated *Xenopus* FoxD4L1 proteins. Red lines denote the short β-strand, and the blue ribbon denotes a 1.7 turn α-helix predicted to form by the 6 alanine residues. Dashes over the aspartic (D) and glutamic (E) acid residues indicate negative charges.

**Figure 9:**
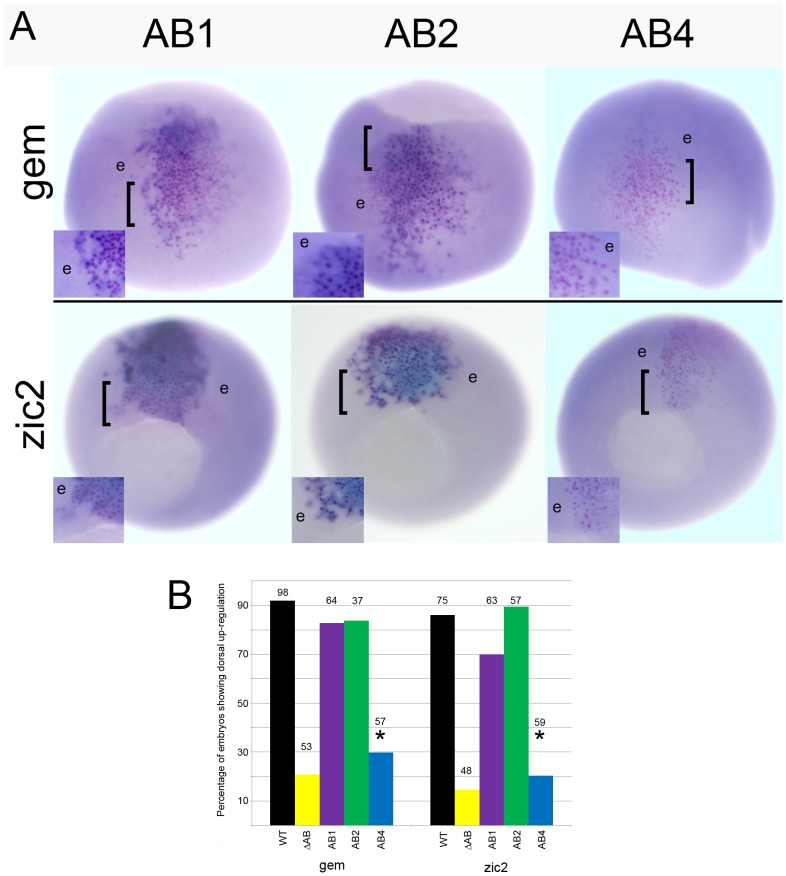
The ability to up-regulate *gem* and *zic2* is lost in the AB4 mutant. (A) The FoxD4L1-AB mutant expressing clones, marked by nuclear βGal (pink dots), are located in the neural ectoderm. For AB1 and AB2, the βGal labeled cells are more intensely stained (darker blue) than their neighboring cells (e) expressing endogenous level of *gem* or *zic2*. For AB4, the βGal labeled cells are stained at the same intensity as the neighboring cells (e). Insets are higher magnifications of the clone, the position of which is indicated on the whole embryo by a bracket. For *gem*, images are dorsal views with vegetal pole to the top; for *zic2*, images are vegetal views with dorsal to the top. (B) The percentage of embryos in which the FoxD4L1-AB mutants caused up-regulation of *gem* or *zic2* in the dorsal neural ectoderm. The data for the ΔAB mutant (14aa deletion in Fig. 8A) is shown for comparison. Numbers above each bar indicates sample size; * indicates significant difference from wild type (WT) at the p<0.001 level. Data for WT and ΔAB are from [39].

We previously reported that the ability of *Xenopus* FoxD4L1 to up-regulate *gem* and *zic2* requires a 14 amino acid stretch, called the acidic blob (AB; aa21–34, Figure 8), within the N-terminal region of the protein [39]. Psipred and Porter predicted the N-terminal region of *Xenopus* FoxD4L1 to be random coil and disordered, but Porter additionally predicted a short β-strand (aa 26–29, IDIL) within the AB (Table 1). CLUSTALW alignment of mouse, human, fish and frog FoxD4/FoxD4L1 proteins demonstrated that this sequence is conserved (IDVL/IDIV/IDIL; Figure 8A), and Porter predicts it to form a short β-strand in all five proteins (Table 1). To test whether this site might serve as a “folding center” in a region that is predicted to be random coil and disordered, we: 1) deleted IDILGE (aa26–31; AB1 mutation); 2) replaced IDILGE with 6 alanine residues to disrupt the β-strand formation since alanines have higher propensity to form α-helices (AB4 mutation); and 3) replaced the highly conserved IDIL with 6 alanines to disrupt the β-strand and change the spacing of the two acidic regions (AB2 mutation) (Figure 8B). Western blot analysis showed that all three AB mutants were expressed as well as wild-type FoxD4L1 (Figure 4B). AB1- and AB2-expressing clones located in the neural plate up-regulated *gem* and *zic2* expression above endogenous levels (Figure 9A) at frequencies statistically equivalent to wild-type FoxD4L1 (Figure 9B), indicating that they retain wild-type protein function. In contrast, AB4-expressing clones were significantly impaired in their ability to up-regulate these genes, and at frequencies equivalent to deleting the entire AB (Figure 9A, B). As described above for the wild-type, myc-tagged FoxD4L1 protein, the myc-tagged AB4 mutant protein is found in both the cytoplasm and nucleus (Figure 6C). To ascertain with confidence that the AB4 immunofluorescence was intra-nuclear, confocal microscopy using signature spectral curve analysis of nuclear DAPI staining and immunofluorescence of a myc-tagged version of AB4 protein, as described for the GARP mutant, was performed. The presence of single pixels containing both DAPI and Alexa Fluor 488 signatures after removal of the autofluorescence signature demonstrated that the loss of functionality was not due to impaired access to the nucleus (Figure 6C). These results demonstrate that activation of target neTF genes likely requires a flexible structure separating two acidic domains.

**Figure 10:**
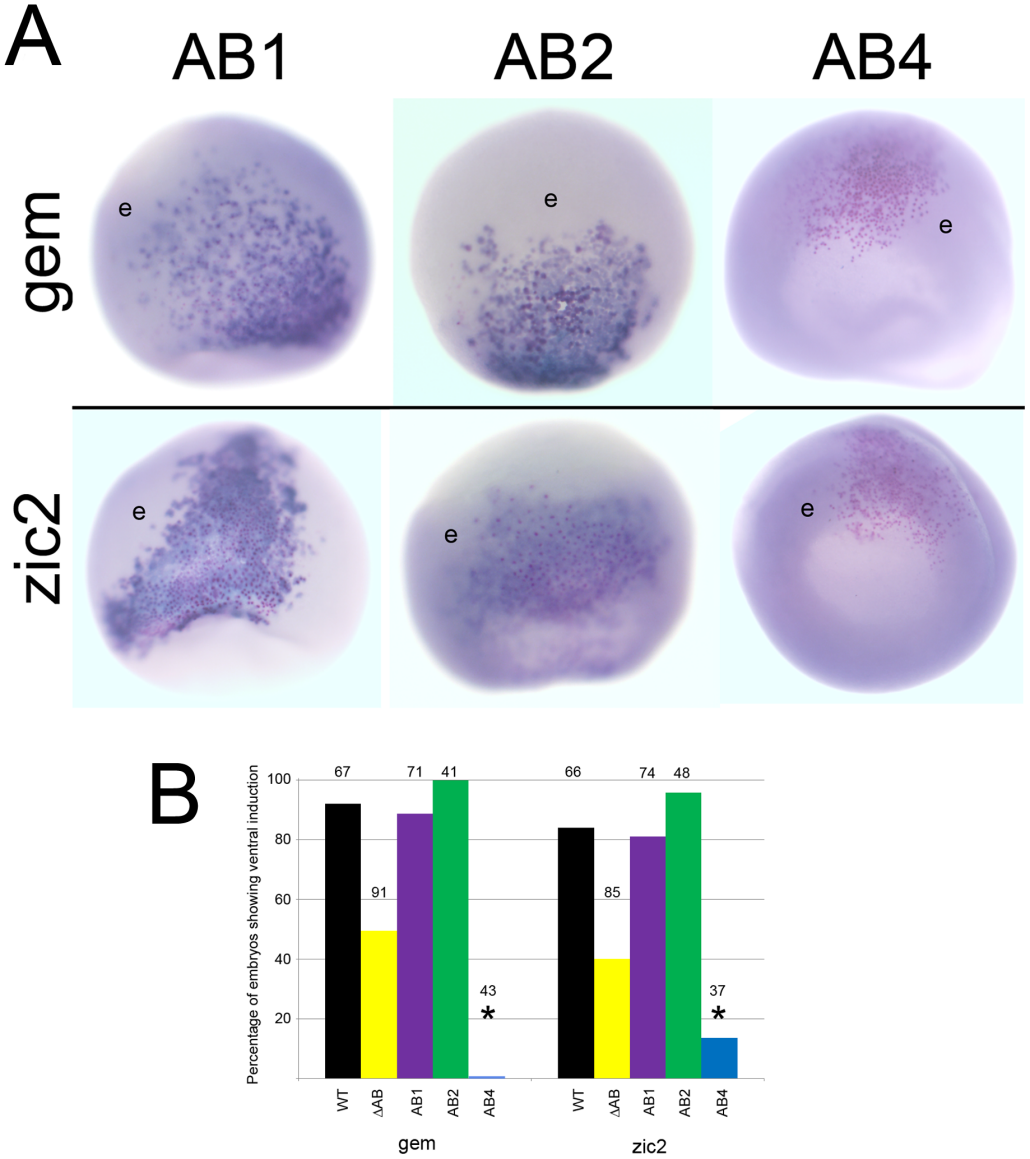
The ability to ectopically induce *gem* and *zic2* is lost in the AB4 mutant. (A) Ventral ectopic expression of *gem* and *zic2* after injection of each FoxD4L1-AB mutant mRNAs into an epidermal precursor blastomere. Clones are indicated by βGal-positive pink dots. In AB1 and AB2 clones, most cells exhibit a high level of expression (dark blue stain), compared to neighboring cells showing endogenous expression levels (e). Cells in the AB4 clones do not express the genes at levels above endogenous (e). gem-AB1, zic2-AB1, and zic2-AB2 are ventral views with animal cap to the bottom; gem-AB2, gem-AB4, zic2-AB4 are animal cap views. (B) The percentage of embryos in which the FoxD4L1-AB mutants induced *gem* or *zic2* expression in the ventral ectoderm. Labeling is as in 9B.

We also tested the AB mutants in a ventral induction assay. We previously showed that ectopically expressing wild-type FoxD4L1 in a ventral epidermal precursor blastomere could convert its progeny to a neural fate, as measured by the cell-autonomous ectopic expression of *gem* and *zic2* [37]. Furthermore, deleting the entire AB impaired this ectopic induction [39]. We performed the same assay with the AB mutants and found that the AB1 and AB2 mutants were as effective in the ventral induction of neTFs as wild type FoxD4L1 (Figure 10A, B). In contrast, the AB4 mutant never induced *gem* and rarely induced *zic2* in the ventral epidermis. Thus, the same structural conformation that up-regulates these neTF genes in the neural ectoderm also is required for their ectopic induction in the epidermal lineage.

## Discussion

FoxD4/FoxD4L1 is expressed in the developing nervous system, and in *Xenopus* plays a key role in expanding the neural plate [27], [32], [34]. This is accomplished by both up-regulating neTF genes that maintain an immature neural ectoderm and down-regulating neTF genes that promote neural differentiation [37]. A structure-function analysis demonstrated that an interaction with the Grg4 (Groucho) co-repressor via an Eh-1 motif in the C-terminal region contributes to FoxD4L1’s down-regulation of some *sox*, *zic* and *irx* genes [39]. However, this interaction did not account for all of the repression. Our study also showed that within the N-terminal region a 14-amino acid acidic region comprises the transactivation domain [39], consistent with an activating role for highly acidic regions in other transcription factors [60], [61]. Because the dual functionality of this protein has an important impact on the earliest steps of neural development, i.e., maintaining the nascent neural ectoderm in a proliferative, immature state so that it can be expanded, we sought to uncover additional motifs or secondary structure that provide additional repressive function or are required for transactivation of target genes.

### A predicted α-helical structure in the C-terminus contributes to the repressive activity of FoxD4L1

Analysis of the FoxD4/FoxD4L1 amino acid sequences across several vertebrates revealed potential sites for protein-protein interactions in the C-terminus, some in the proline-rich region between the DNA binding domain and the well characterized Eh-1 motif that can bind Grg proteins (e.g., Motif 2), and some downstream of the Eh-1 motif (e.g., Motifs 3, 6, 8, FH2). Based on our previous deletions, we predicted that motifs located downstream of the Eh-1 motifs would be the most likely to contribute to repressive activity. Since the various programs consistently predicted FoxD4/FoxD4L1 to be random coil and disordered, and disordered proteins often are dynamically flexible so they can form conformations that facilitate binding to multiple protein and/or DNA targets [62], we hypothesized that the putative α-helical/Motif 6 region at the extreme C-terminus would be functionally important. Our study confirmed functionality of this region by demonstrating that a single amino acid substitution predicted to disrupt an α-helical structure significantly reduces transcriptional repression. Mutations hypothesized to merely destabilize an α-helical structure, however, were tolerated without loss of function. Future experiments should functionally test the other motifs identified in the C-terminus, in particular the highly conserved FH2 motif, to determine if they also contribute to the repressive activity of FoxD4/FoxD4L1 proteins.

We analyzed other FoxD proteins to determine if the arrangement of a Grg/Groucho binding domain followed by a predicted α-helical region is conserved (Table 1). In *Xenopus*, Fox D1, FoxD3 and FoxD4L1 all contain this arrangement, whereas FoxD2 is not predicted to contain an α-helix. Mouse FoxD3, mouse FoxD4, human FoxD4 and human FoxD4L1 each are predicted to contain this arrangement, suggesting a functional importance. Interestingly, in sea urchin, the FoxQ2 protein rather than a FoxD protein, is essential for neural fate [63]; we found an Eh-1 domain (FSIENL, aa4–9) followed by a predicted α-helix (Psipred >70% confidence; MKVLVQQE, aa 29–36) in the N-terminus. Likewise, we found predicted α-helical regions in *Xenopu*s and mouse FoxA1 and FoxA2 proteins located in close proximity to the Eh-1 motif in the C-terminus (Table 2). Because in mouse these two proteins repress target genes via an interaction with Grg that subsequently binds to acetylated histone to compact nucleosomes [41], this secondary structure may facilitate these interactions. Thus, our work uniquely identifies a functionally important putative α-helical region separated from a Grg/Groucho binding domain in several chordate Fox transcriptional repressor proteins, suggesting that this is a critical structural relationship.

**Table 2:** Predicted C-terminal structures in FoxA proteins.

		Psipred	Porter
	Eh-1	α-helix	α-helix
Mouse FoxA1	aa 396–402	aa 415–420	aa 414–421
Xenopus FoxA1	aa 356–362	aa 374–381	random coil
Mouse FoxA2	aa 377–383	random coil	aa 401–410
Xenopus FoxA2	aa 351–357	aa 379–384	aa 383–386

Legend: The C-terminus of each FoxA protein contains a conserved Eh-1 motif at the amino acid (aa) location indicated. At locations downstream of this motif, the proteins are predicted to either be random coil or to form an α-helical structure at the indicated locations.

### Flexibility within the AB likely accounts for the transactivation activity of FoxD4L1

Analysis of the N-terminal region of FoxD4/FoxD4L1 across human, mouse, fish and frog predicted a random coil and disordered structure except in the AB domain (Table 1). Since our previous work identified the AB as responsible for target neTF gene up-regulation [39], we sought to define which amino acids within this 14 residue stretch are critical for transactivation. Our analyses predicted a four amino acid β-strand in the frog sequence that separates two clusters of acidic residues (Figure 8A). Surprisingly, neither deleting the β-strand (IDIL) nor replacing it with a putative short α-helical structure diminished activation of target neTF genes as long as the glycine residue was intact. We predict that target gene activation relies on the two regions of acidic residues coming into close proximity, via flexibility at the glycine residue (Figure 8B). In AB1, removal of the β-strand brings the two small acidic regions (DEEDE, aa21–25; EDD, aa31–33) next to each other, and in AB2 the remaining glycine provides sufficient flexibility to bring the acidic regions together. However, removing the glycine rendered the protein nearly incapable of activating target neTF genes in either the neural ectoderm, where they are endogenously expressed, or in the epidermis, where they can be induced by the wild-type protein. These results suggest that target gene activation relies on a structure that allows two regions of acidic residues (aa21–25 and aa31–33) to come into close proximity (Figure 8B).

The IDIL sequence found in *Xenopus* FoxD4L1 is highly conserved in other FoxD proteins in mouse, human and frog (Table 1; IDVV, IDVL). For all except *Xenopus* FoxD1, Porter predicts these to form a β-strand, and in all proteins a glycine residue follows this sequence, either immediately or within 5 residues. In all of these FoxD proteins the IDIL/IDVV/IDVL sequence is flanked by acidic residues. Thus, we predict that the functional importance of two acidic regions separated by polypeptide flexibility via an intervening glycine residue is likely conserved across species.

### The identified functional domains are highly conserved

These analyses identify unique domains in the FoxD4/FoxD4L1 proteins that rely on secondary structure in addition to specific amino acid motifs for the protein to function as both a transcriptional activator and repressor. Elucidating the molecular mechanisms by which this transcription factor interacts with the DNA and other proteins is of fundamental importance because its targets regulate the critical processes of expanding the nascent neural ectoderm and initiating the onset of neural differentiation. Because the subtle predicted structures described herein are highly conserved, the results are likely to apply to the function of the FoxD4/FoxD4L1 proteins in many other animals, including humans. Further, other FoxD sub-family proteins contain similar structures (Table 1), suggesting that these features are functionally conserved across the sub-family. This is the first report of the functional significance of two of these newly identified motifs/structural domains. Identifying potential interacting partners for each predicted motif and secondary structure, and unraveling how they affect protein function are important next steps.

## Supporting Information

Figure S1 Multiple sequence alignments of FoxD4L1 of fish and amphibians. The sequence alignment shows the consensus sequences, conservation and the quality of sequence alignment. The sequences alignments were analyzed by software Jalview 2.8 [66].
(TIF)

Figure S2 Ten statistically significant C-terminal motifs identified with the expectation-maximization algorithm implemented in the MEME program in FoxD4L1 of fish and amphibians [55]. Those indicated by 9–10 sites are found in both frog and fish, whereas those indicted by only 5 sites are amphibian-specific.
(TIF)

Figure S3 Multiple sequence alignments of FoxD4/FoxD4L1 of amphibians and mammals. The sequence alignment shows the consensus sequences, conservation and the quality of sequence alignment. The sequences alignments were analyzed by software Jalview 2.8 [66].
(TIF)

Figure S4 Ten statistically significant C-terminal motifs identified with the expectation-maximization algorithm implemented in the MEME program in FoxD4/FoxD4L1 of mammals and amphibians [55].
(TIF)

Figure S5 A wheel model of the Leucine (Leu) repeating region of *Xenopus* FoxD4L1A (aa 313–330) indicated that it may form an amphipathic α-helical structure.
(TIF)

Figure S6 Prediction of secondary structure of *Xenopus* FoxD4L1A using the Network Protein Sequence Analysis server. As a comparison, the secondary structure determined in the crystal structure studies in FoxD3 (Genesis/Hfh2) of the winged helix DNA-binding domain, accession number: 2HFH_A. α-helical structures are shown in underlined bold and β-sheets are in underlined italic bold [59].
(DOC)

Table S1 Gene and protein accession numbers for vertebrate FoxD4L1.
(XLSX)
