# Correction: Conserved Structural Domains in FoxD4L1, a Neural Forkhead Box Transcription Factor, Are Required to Repress or Activate Target Genes

**DOI:** 10.1371/annotation/b1f1b311-f2ec-4c09-870f-5771fcbdc3ed

**Published:** 2013-04-22

**Authors:** Steven L. Klein, Karen M. Neilson, John Orban, Sergey Yaklichkin, Jennifer Hoffbauer, Kathy Mood, Ira O. Daar, Sally A. Moody

There were errors in the Funding section. The correct funding information is as follows: 

This research was supported in part by facilitating funds from the George Washington University School of Medicine (SAM), National Science Foundation grant MCB-1121711 (SAM), and the Intramural Research Program of the National Institutes of Health, National Cancer Institute project number ZIA BC01000617 (IOD). Imaging was supported by NIH NICHD 2P30 HD040677 and NIH NCRR 1S10RR025565-01. Dr Klein’s efforts were supported by the National Science Foundation while working at the Foundation. Any opinions, findings, and conclusions or recommendations expressed in this material are those of the authors and do not necessarily reflect the views of the National Science Foundation. The funders had no role in study design, data collection and analysis, decision to publish, or preparation of the manuscript.

