# Supplementary figures and images for: Conserved Structural Domains in FoxD4L1, a Neural Forkhead Box Transcription Factor, Are Required to Repress or Activate Target Genes
Source: PLoS One. 2013 Apr 16;8(4):e61845. doi: 10.1371/journal.pone.0061845 (PMC3627651; doi:10.1371/journal.pone.0061845)

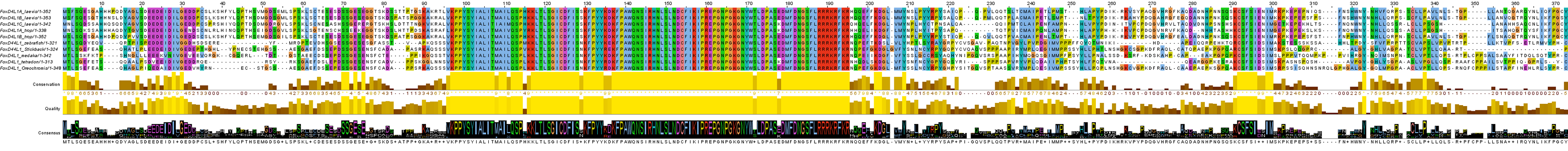

Supplement: Figure S1 — Multiple sequence alignments of FoxD4L1 of fish and amphibians. The sequence alignment shows the consensus sequences, conservation and the quality of sequence alignment. The sequences alignments were analyzed by software Jalview 2.8 [66]. (TIF) [file pone.0061845.s001.tif]

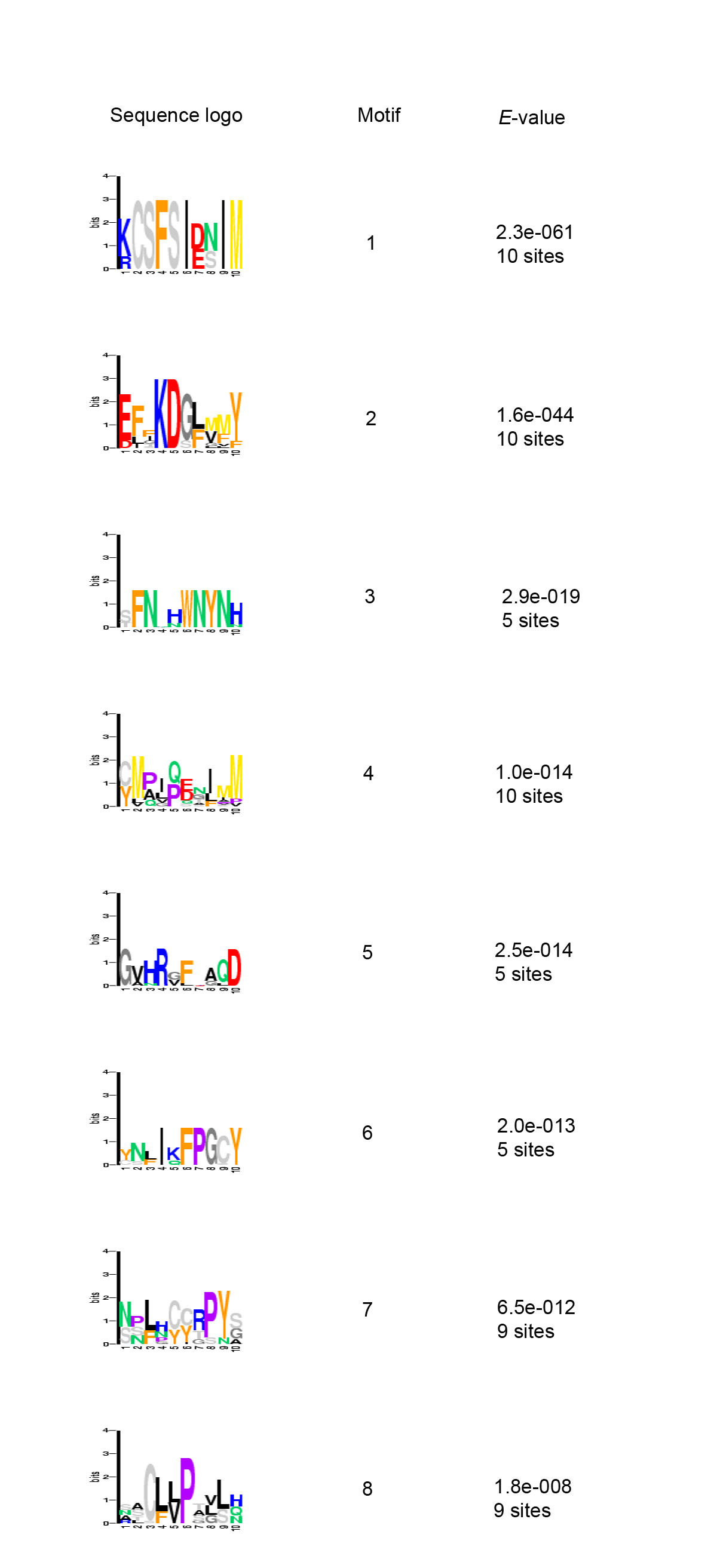

Supplement: Figure S2 — Ten statistically significant C-terminal motifs identified with the expectation-maximization algorithm implemented in the MEME program in FoxD4L1 of fish and amphibians [55]. Those indicated by 9–10 sites are found in both frog and fish, whereas those indicted by only 5 sites are amphibian-specific. (TIF) [file pone.0061845.s002.tif]

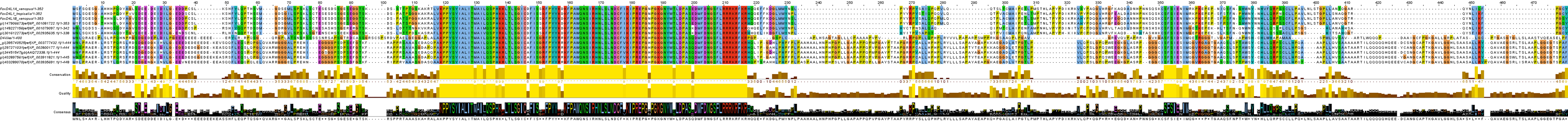

Supplement: Figure S3 — Multiple sequence alignments of FoxD4/FoxD4L1 of amphibians and mammals. The sequence alignment shows the consensus sequences, conservation and the quality of sequence alignment. The sequences alignments were analyzed by software Jalview 2.8 [66]. (TIF) [file pone.0061845.s003.tif]

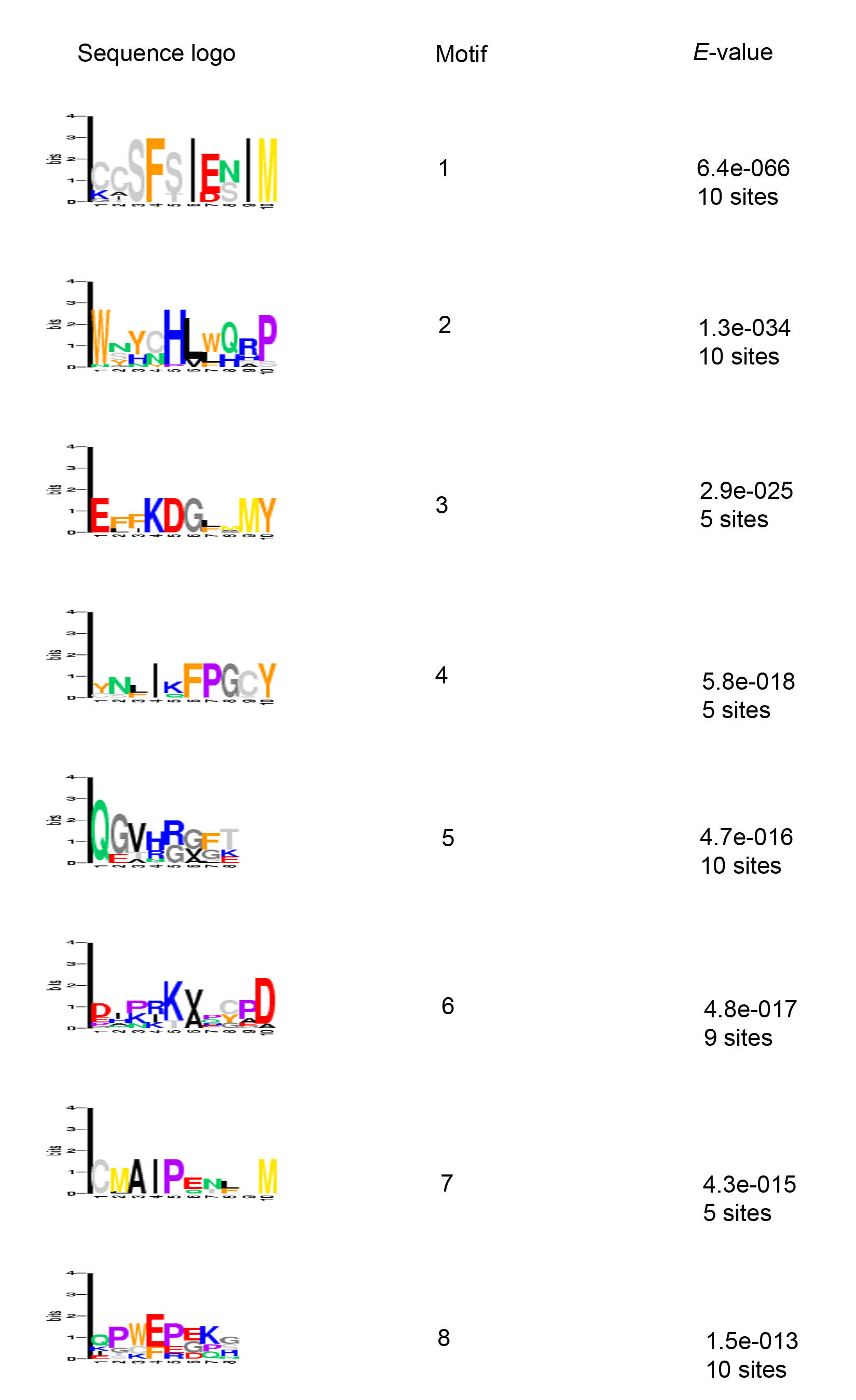

Supplement: Figure S4 — Ten statistically significant C-terminal motifs identified with the expectation-maximization algorithm implemented in the MEME program in FoxD4/FoxD4L1 of mammals and amphibians [55]. (TIF) [file pone.0061845.s004.tif]

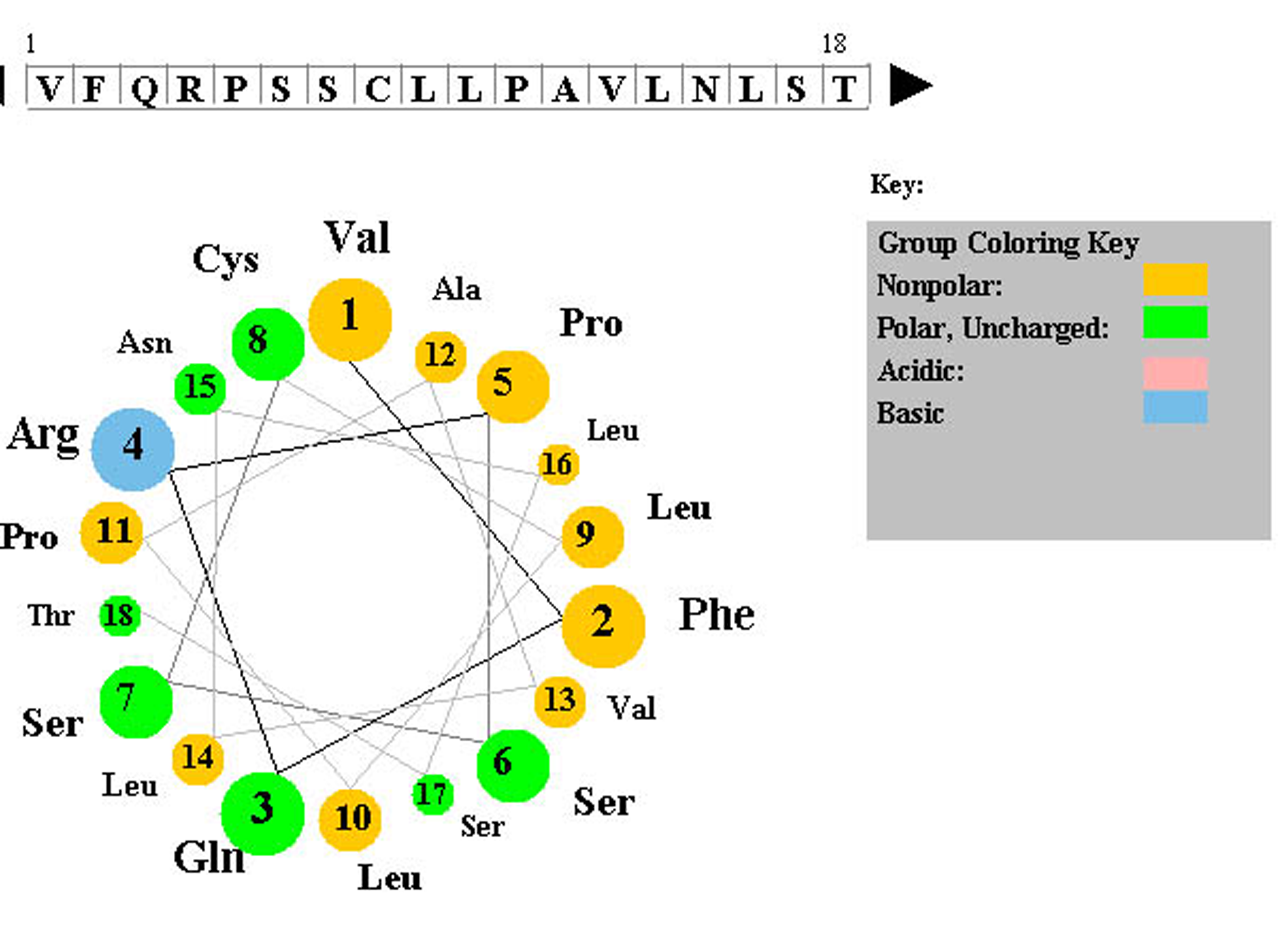

Supplement: Figure S5 — A wheel model of the Leucine (Leu) repeating region of Xenopus FoxD4L1A (aa 313–330) indicated that it may form an amphipathic α-helical structure. (TIF) [file pone.0061845.s005.tif]
