# Supplementary material for: Conserved Structural Domains in FoxD4L1, a Neural Forkhead Box Transcription Factor, Are Required to Repress or Activate Target Genes
Source: PLoS One. 2013 Apr 16;8(4):e61845. doi: 10.1371/journal.pone.0061845 (PMC3627651; doi:10.1371/journal.pone.0061845)
Supplement: Figure S6 — Prediction of secondary structure of Xenopus FoxD4L1A using the Network Protein Sequence Analysis server. As a comparison, the secondary structure determined in the crystal structure studies in FoxD3 (Genesis/Hfh2) of the winged helix DNA-binding domain, accession number: 2HFH_A. α-helical structures are shown in underlined bold and β-sheets are in underlined italic bold [59]. (DOC) [file pone.0061845.s006.doc]

10 20 30 40 50 60 70

| | | | | | |

UNK_24850 MSFSQESGAHHHPQDYAGLSDEEDEIDILGEDDPCSLKSHFYLQPTHSVMGDSEMLSPSKLSCTESESDS

DSC cccccccccccccccccccchhhhhhhcccccccccccceeeeccccccccccccccccccceeeccccc

HNNC cccccccccccccccccccccchhheeeecccccccccceeecccccccccccccccccccccccccccc

MLRC ccccccccccccccccccccccccceeeecccccccccceeeeccccccccccccccccceecccccccc

PHD ccccccccccccccccccccchhhhhhhccccccccccccceeccccccccccccccccccccccccccc

Predator cccccccccccccccccccccccccccccccccccccccccccccccccccccccccccccccccccccc

Sec.Cons. cccccccccccccccccccccchhh???ccccccccccceeeeccccccccccccccccccccccccccc

80 90 100 110 120 130 140

| | | | | | |

UNK_24850 SGESEGGTSKDSSTTPTGSKAKRTLVKPPYS**YIALITMAIL**QSPH***KKLT*LSGICDFISS**K**FPYYKDKFPA**

DSC cccccccccccccccccchhhhhcccccccchhhhhhhhhhhhhhhhcchhhhhhhhhcccccccccccc

HNNC ccccccccccccccccccccccccccccchhhhhhhhhhhhhcccceeeecchhhhhhcccccccccchc

MLRC cccccccccccccccccccccceeeeccccchhhehhhhhhcccccceeecchhhhhhcccccccccccc

PHD ccccccccccccccccccccccccccccchhhhhhhhhhhhhccccccechhhhhhhhhcccccccccch

Predator ccccccccccccccccccccccccccccccchhhhhhhhhhccccccccccccccccccccccccccccc

Sec.Cons. ccccccccccccccccccccccccccccccchhhhhhhhhhhcccccce?cchhhhhhcccccccccccc

150 160 170 180 190 200 210

| | | | | | |

UNK_24850 W**QNSIRHNLSLND**C***FIKI***PREPGNPGKG***NYWTL***DPASEDMFDNGSFLRRRKRFKRHQQEFFKDGLMMYNS

DSC hhhhhcccccccccchhcccccccccccceeecccchhhhhhhhhhhhhhhhhhhhhhhhhhhhhhhccc

HNNC hhhheeeccccchhhheccccccccccccceeccccchchhcchhhhhhhhhhhhhhhhhhhccheeecc

MLRC chhhhcccccccceeeeeccccccccccceeeecccccccccccchhhhhhhhhhcchhhhhhcceeccc

PHD hhcccccccccchhhhcccccccccccccceeccccchhhccccccehhhhhhcchhhhhhhhccccccc

Predator ccccccccccccccceeeeccccccccccccccccccccccccccchhhhhhhhhhhhhhhcccceeecc

Sec.Cons. hhhh?cccccccc??heccccccccccccceeccccchchccccchhhhhhhhhhhhhhhhhhcceeccc

220 230 240 250 260 270 280

| | | | | | |

UNK_24850 LPYYRPYSAIQPQPVLQQTSLTCMAIPETLPMSTHLAPYPDIKRKVSYPAQGVHRGFKAQDADNHPNNSQ

DSC ccccccccccccccccccchhhhhccccccccccccccccchhhhhcccchhhhhhhhhhcccccccccc

HNNC cccccccccccccccccccceeeeecccccccccccccccccchhccccccccccccccccccccccccc

MLRC cccccccccccccccccccceeeeecccccccccccccccchcccccccccccccccccccccccccccc

PHD cccccccccccccccccccccccccccccccccccccccccccccccccccccccccccccccccccccc

Predator ccccccccccccccccchhhhhhhcccccccccccccccccccccccccccccccccccccccccccccc

Sec.Cons. cccccccccccccccccccc????cccccccccccccccccccccccccccccccccccccccccccccc

290 300 310 320 330 340 350

| | | | | | |

UNK_24850 SKCSFSIENIMRKPKEPEPNIQSFNSHWNYNHVFQRPSSCLLPAVLNLSTGPLLANTQGARQYNLIQFPG

DSC ccccccchhhhhcccccccccccccccccccccccccccccccceecccccccccccchhhhhhhhcccc

HNNC ccceeeehhhhccccccccccccccccccccccccccchhhhhhhhccccccccccccchchceeeeccc

MLRC cccccchhhhhhccccccccccccccccccceecccccccccceeeeccccceecccccccceeeeeecc

PHD cccccccccccccccccccccccccccccccccccccccccccccccccccccccccccccccccccccc

Predator ccchhhhhhhhccccccccccccccccccceeeeecccccccccceeecccccccccccchhhhhccccc

Sec.Cons. cccccc?hhhhccccccccccccccccccccccccccccccccc?eccccccccccccccch????cccc

UNK_24850 CY

DSC cc

HNNC cc

MLRC cc

PHD cc

Predator cc

Sec.Cons. cc

Sequence length : 352

DSC :

Alpha helix (Hh) : 108 is 30.68%

3_10_  helix (Gg) : 0 is 0.00%

Pi helix (Ii) : 0 is 0.00%

Beta bridge (Bb) : 0 is 0.00%

Extended strand (Ee) : 12 is 3.41%

Beta turn (Tt) : 0 is 0.00%

Bend region (Ss) : 0 is 0.00%

Random coil (Cc) : 232 is 65.91%

Ambigous states (?) : 0 is 0.00%

Other states : 0 is 0.00%

HNNC :

Alpha helix (Hh) : 70 is 19.89%

3_10_  helix (Gg) : 0 is 0.00%

Pi helix (Ii) : 0 is 0.00%

Beta bridge (Bb) : 0 is 0.00%

Extended strand (Ee) : 33 is 9.38%

Beta turn (Tt) : 0 is 0.00%

Bend region (Ss) : 0 is 0.00%

Random coil (Cc) : 249 is 70.74%

Ambigous states (?) : 0 is 0.00%

Other states : 0 is 0.00%

MLRC :

Alpha helix (Hh) : 42 is 11.93%

3_10_  helix (Gg) : 0 is 0.00%

Pi helix (Ii) : 0 is 0.00%

Beta bridge (Bb) : 0 is 0.00%

Extended strand (Ee) : 48 is 13.64%

Beta turn (Tt) : 0 is 0.00%

Bend region (Ss) : 0 is 0.00%

Random coil (Cc) : 262 is 74.43%

Ambigous states (?) : 0 is 0.00%

Other states : 0 is 0.00%

PHD :

Alpha helix (Hh) : 53 is 15.06%

3_10_  helix (Gg) : 0 is 0.00%

Pi helix (Ii) : 0 is 0.00%

Beta bridge (Bb) : 0 is 0.00%

Extended strand (Ee) : 6 is 1.70%

Beta turn (Tt) : 0 is 0.00%

Bend region (Ss) : 0 is 0.00%

Random coil (Cc) : 293 is 83.24%

Ambigous states (?) : 0 is 0.00%

Other states : 0 is 0.00%

Predator :

Alpha helix (Hh) : 45 is 12.78%

3_10_  helix (Gg) : 0 is 0.00%

Pi helix (Ii) : 0 is 0.00%

Beta bridge (Bb) : 0 is 0.00%

Extended strand (Ee) : 15 is 4.26%

Beta turn (Tt) : 0 is 0.00%

Bend region (Ss) : 0 is 0.00%

Random coil (Cc) : 292 is 82.95%

Ambigous states (?) : 0 is 0.00%

Other states : 0 is 0.00%

Sec.Cons. :

Alpha helix (Hh) : 50 is 14.20%

3_10_  helix (Gg) : 0 is 0.00%

Pi helix (Ii) : 0 is 0.00%

Beta bridge (Bb) : 0 is 0.00%

Extended strand (Ee) : 11 is 3.12%

Beta turn (Tt) : 0 is 0.00%

Bend region (Ss) : 0 is 0.00%

Random coil (Cc) : 274 is 77.84%

Ambigous states (?) : 17 is 4.83%

Other states : 0 is 0.00%


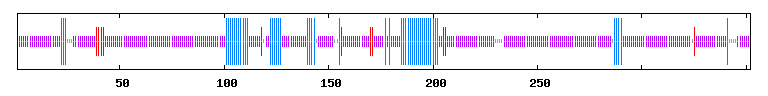


PREDATOR parameters :

Secondary structure data : dssp

View prediction result file in MPSA : [[67AE8D0071E0.DSC](http://npsa-pbil.ibcp.fr/cgi-bin/npsa_mpsaview.pl?fn=/tmp/67ae8d0071e0.dsc&gui=mpsa)] [[67AE8D0071E0.HNN](http://npsa-pbil.ibcp.fr/cgi-bin/npsa_mpsaview.pl?fn=/tmp/67ae8d0071e0.hnn&gui=mpsa)] [[67AE8D0071E0.MLR](http://npsa-pbil.ibcp.fr/cgi-bin/npsa_mpsaview.pl?fn=/tmp/67ae8d0071e0.mlr.mpsa&gui=mpsa)] [[67AE8D0071E0.PHD](http://npsa-pbil.ibcp.fr/cgi-bin/npsa_mpsaview.pl?fn=/tmp/67ae8d0071e0.phd&gui=mpsa)] [[67AE8D0071E0.PREDA](http://npsa-pbil.ibcp.fr/cgi-bin/npsa_mpsaview.pl?fn=/tmp/67ae8d0071e0.preda&gui=mpsa)] 
View prediction result file in ANTHEPROT : [[67AE8D0071E0.DSC](http://npsa-pbil.ibcp.fr/cgi-bin/npsa_mpsaview.pl?fn=/tmp/67ae8d0071e0.dsc&gui=antheprot)] [[67AE8D0071E0.HNN](http://npsa-pbil.ibcp.fr/cgi-bin/npsa_mpsaview.pl?fn=/tmp/67ae8d0071e0.hnn&gui=antheprot)] [[67AE8D0071E0.MLR](http://npsa-pbil.ibcp.fr/cgi-bin/npsa_mpsaview.pl?fn=/tmp/67ae8d0071e0.mlr.mpsa&gui=antheprot)] [[67AE8D0071E0.PHD](http://npsa-pbil.ibcp.fr/cgi-bin/npsa_mpsaview.pl?fn=/tmp/67ae8d0071e0.phd&gui=antheprot)] [[67AE8D0071E0.PREDA](http://npsa-pbil.ibcp.fr/cgi-bin/npsa_mpsaview.pl?fn=/tmp/67ae8d0071e0.preda&gui=antheprot)]
